# Genetic and functional evaluation of the role of CXCR1 and CXCR2 in susceptibility to visceral leishmaniasis in north-east India

**DOI:** 10.1186/1471-2350-12-162

**Published:** 2011-12-15

**Authors:** Sanjana Mehrotra, Michaela Fakiola, Joyce Oommen, Sarra E Jamieson, Anshuman Mishra, Medhavi Sudarshan, Puja Tiwary, Deepa Selvi Rani, Kumarasamy Thangaraj, Madhukar Rai, Shyam Sundar, Jenefer M Blackwell

**Affiliations:** 1 Institute of Medical Sciences, Banaras Hindu University, Varanasi, OS 221 005, India; 2 Telethon Institute for Child Health Research, Centre for Child Health Research, The University of Western Australia, Subiaco, Western Australia, Australia; 3 Cambridge Institute for Medical Research and Department of Medicine, University of Cambridge School of Clinical Medicine, Cambridge, UK; 4 Centre for Cellular and Molecular Biology, Hyderabad, India

## Abstract

**Background:**

IL8RA and IL8RB, encoded by *CXCR1 *and *CXCR2*, are receptors for interleukin (IL)-8 and other CXC chemokines involved in chemotaxis and activation of polymorphonuclear neutrophils (PMN). Variants at *CXCR1 *and *CXCR2 *have been associated with susceptibility to cutaneous and mucocutaneous leishmaniasis in Brazil. Here we investigate the role of *CXCR1/CXCR2 *in visceral leishmaniasis (VL) in India.

**Methods:**

Three single nucleotide polymorphisms (SNPs) (rs4674259, rs2234671, rs3138060) that tag linkage disequilibrium blocks across *CXCR1/CXCR2 *were genotyped in primary family-based (313 cases; 176 nuclear families; 836 individuals) and replication (941 cases; 992 controls) samples. Family- and population-based analyses were performed to look for association between *CXCR1/CXCR2 *variants and VL. Quantitative RT/PCR was used to compare CXCR1/CXCR2 expression in mRNA from paired splenic aspirates taken before and after treatment from 19 VL patients.

**Results:**

Family-based analysis using FBAT showed association between VL and SNPs *CXCR1*_rs2234671 (Z-score = 2.935, *P *= 0.003) and *CXCR1*_rs3138060 (Z-score = 2.22, *P *= 0.026), but not with *CXCR2*_rs4674259. Logistic regression analysis of the case-control data under an additive model of inheritance showed association between VL and SNPs *CXCR2*_rs4674259 (OR = 1.15, 95%CI = 1.01-1.31, *P *= 0.027) and *CXCR1*_rs3138060 (OR = 1.25, 95%CI = 1.02-1.53, *P *= 0.028), but not with *CXCR1*_rs2234671. The 3-locus haplotype T_G_C across these SNPs was shown to be the risk haplotype in both family- (TRANSMIT; *P *= 0.014) and population- (OR = 1.16, *P *= 0.028) samples (combined *P *= 0.002). CXCR2, but not CXCR1, expression was down regulated in pre-treatment compared to post-treatment splenic aspirates (*P *= 0.021).

**Conclusions:**

This well-powered primary and replication genetic study, together with functional analysis of gene expression, implicate CXCR2 in determining outcome of VL in India.

## Background

Visceral leishmaniasis (VL), also known as Kala-azar, is a life-threatening disease caused by protozoans belonging to *Leishmania donovani *complex. Population based epidemiological surveys suggest that 80-90% of individuals infected with *L. donovani *show no clinical symptoms [1-3]. Familial clustering, and the range of clinical outcomes from asymptomatic to fatal disease within and between ethnic groups sharing similar risk factors, support a contribution of host genotype to susceptibility [4-8]. Resistance to VL, as determined by a positive antigen-specific DTH response without clinical symptoms, is ~80% heritable in family-based studies [9]. Genetic variability in ability to mount an innate immune response, such as in the influx of polymorphonuclear neutrophils (PMN) during initial hours of infection, could be important in innate killing of the parasite [10], and in providing the cytokine environment in which parasite-specific T cells are primed [11]. Pro-inflammatory responses elicited by PMN as part of the response to the bite of the sand fly vector are important in initiation of infection [12]. The arrival and maintenance of infiltrating cells at bite sites is thought to be mediated by sand fly-derived factors that mimic a tissue damage signal and/or activate chemokine/chemokine receptor pathways [13-15]. Recently [16] we examined the role of polymorphisms at *CXCR1 *and *CXCR2*, which act as receptors for CXC chemokines that attract PMN to inflammatory sites, in determining susceptibility to cutaneous forms of leishmaniasis caused by infection with *L. braziliensis*. We found that cutaneous (CL) and mucocutaneous (ML) forms of disease were associated with opposing alleles for SNP rs2854386 at *CXCR1*. Studies in mice [17] show that selective depletion of PMN has a dramatic effect on the course of infection with *L. donovani*. Here we use genetic and functional approaches to evaluate the role of PMN in VL caused by *L. donovani *in humans through analysis of the receptors CXCR1, which is a specific receptor for IL-8 (= CXCL8), and CXCR2, which is promiscuous in binding a variety of CXC chemokines (CXCL-1, 2, 3, 5, 6, 7) in addition to CXCL8.

## Methods

The study was conducted in the district of Muzaffarpur in Bihar State, India, where VL is highly endemic. Diagnosis of VL was made on the basis of clinical, parasitological and serological criteria as described [18,19]. Further epidemiological and demographic details relating to the study samples and study site are described elsewhere [20,21]. Informed written consent in Hindi was obtained from all participating individuals and from parents of children under 18 years old. Approval for the study was provided by the Ethical Committee of the Institute of Medical Sciences, Banaras Hindu University, Varanasi, India. Collection of families was undertaken between 2004 and 2006 while for the case-control study collection was undertaken during 2009-2010. Table 1 provides details of primary and replication samples, indicating the number of extended families that are decomposed into nuclear families for genetic analysis. For the family-based primary study, DNA was prepared from buccal swabs by whole genome amplification as described [19]. For the replication case-control study, genomic DNA was extracted from saliva using the Oragene technology (DNA Genotek, Ontario, Canada).

**Table 1:** Baseline characteristics of (A) families for the primary sample of Indian multicase VL families, and (B) the Indian case-control cohorts

(A) Family Structure	Number*
N^o ^families	137

N^o ^nuclear families	176

Nuclear families with 1 affected sib	63

Nuclear families with 2 affected sibs	95

Nuclear families with 3 affected sibs	14

Nuclear families with 4 affected sibs	2

Nuclear families with 5 affected sibs	2

N^o ^affected offspring	313

N^o ^affected parents	63

Total N^o ^affected individuals	394

Total N^o ^individuals	836

**(B) Case-Control Sample**	**Number**

**Cases (no.)**	958

Male	571

Female	387

Mean age at study encounter ± SD (yr)	31.2 ± 16.7

Range	3-73

Mean age at onset of VL ± SD (yr)	26.8 ± 15.3

Religious Group (no.)	

Hindu	850

Muslim	108

**Controls (no.)**	1015

Male	570

Female	445

Mean age at study encounter ± SD (yr)	31.8 ± 15.9

Religious Group (no.)	

Hindu	885

Muslim	130

* Numbers are given for the individuals with DNA available for genotyping

*CXCR1 *and *CXCR2 *lie on a 26 kb region adjacent to each other on human Chromosome 2q35. *CXCR2 *is encoded on the positive strand and lies proximal to *CXCR1 *encoded on the negative strand. DNA samples were genotyped for SNPs (Table 2) *CXCR2 *(rs4674259) and *CXCR1 *(rs3138060) that tag major linkage disequilibrium (LD) blocks (r^2 ^> 0.8; as determined for available data from HapMap populations CEU/JPT/CHB; Additional File 1; Figure S1) across the two genes, as well as the non-synonymous exon 1 SNP rs2234671 in *CXCR1*. For the primary family-based study, SNPs were genotyped in 836 individuals using ABI predesigned Taqman assays (ABI, Mulgrave, Victoria, Australia). For the replication study, SNPs were genotyped using Sequenom iPLEX platform (Sequenom, San Diego, CA, USA) for 2022 individuals comprising 990 cases, 1029 controls (1168 males, 850 females). All 3 SNPs met minimum quality control checks for call rate (> 99.5%) across all individuals, and all were in Hardy Weinberg equilibrium (HWE) in unrelated founders of families and in the control replication sample. Twelve individuals were removed from further analysis where data was missing for two or more SNPs.

**Table 2:** Details of polymorphisms genotyped and the minor allele frequency (MAF) of variants in the Indian study population

SNP Identity	Location	Amino Acid Change	Physical Position^1 ^(bp)	**Alleles**^**2**^	Strand	MAF
CXCR2_rs4674259	5'UTR	-	218991005	T/C	-	0.43

CXCR1_rs2234671	Exon1	S276T	219029108	G/C	-	0.16

CXCR1_rs3138060	Intron1	-	219031500	C/G	-	0.12

^1 ^Physical positions of markers are given according to Build 37.1 of the human genome; ^2^Major > minor alleles for this Indian population; All 3 SNPs were in HWE in both unaffected founders of families and in controls for the replication study.

Family-based allelic association tests were performed within FBAT which is based on the transmission disequilibrium test (TDT) but allows for different genetic model analyses with incomplete parental data [22,23]. Analyses were performed using an additive model and under the null hypothesis of "no linkage and no association". Family-based haplotype TDT was performed using TRANSMIT [24]. Robust tests were performed to take account of multiple trios within some pedigrees (Table 1). Family based TDT power approximations [25] show that the 313 VL trios had 49% power to detect an odds ratio ≥ 1.5 at *P *= 0.01 for markers with MAF ≥ 0.1. Single marker and haplotype association tests for the case-control sample were carried out using logistic regression analysis performed in PLINK [26] under an additive model. Inclusion of caste as a covariate, which we have shown to provide a good surrogate for genetic substructure in genome-wide analyses in this population (unpublished data), was used to take account of population substructure. Religion was also analysed as a covariate. The 1933 individuals (941 cases and 992 controls) which passed quality control had 93.5% power to detect associations with an odds ratio of 1.5 for markers with MAF ≥ 0.1 at *P *= 0.01. Nominal two-tailed *P*-values are presented throughout, i.e. without correction for multiple testing. Application of a strict Bonferroni correction for 3 independent (r^2 ^< 0.8) SNPs provides a significance cut-off of *P *≤ 0.017 (i.e. *P *= 0.05/3). Combined *P*-values using Fisher's Trend Test were calculated using MetaP [27]. LD plots for D' and r^2 ^were generated in Haploview [28].

Splenic biopsies were taken as part of routine diagnostic procedure at the Kala Azar Medical Research Centre, Muzaffarpur, Bihar State, India. Paired pre- (Day-0) and post- (Day-30) treatment splenic samples were collected from 19 VL patients in 5xRNA Later (AMBION Inc., Austin, Texas, USA) during 2009-2010, transported to Varanasi at 4°C and stored at -80°C until RNA was isolated. Details regarding age and sex, splenic parasites and drug administered were recorded for each patient. Total RNA was isolated using RNeasy tissue kit (Qiagen, GmbH, Hilden, Germany) according to the manufacturer's instructions. Sample quality and integrity was assessed by ND-2000 spectrophotometer (Thermo Fischer Scientific, Wilmington, DE, USA) and agarose (Sigma Aldrich Chemicals, St Louis, MO, USA) gel electrophoresis. 1 μg of RNA was reverse transcribed using the High Capacity cDNA synthesis kit (Applied Biosystems, Foster City, CA, USA). Taqman predesigned gene expression assays (CXCR1_Hs00174146_m1 and CXCR2_Hs01011557_m1) were used to perform expression studies (7500 HT Real Time PCR system, Applied Biosystems, Foster City CA, USA) with 18S rRNA (P/N 4319413E) being used as an endogenous control to normalize the expression data. No RT and no template controls were included in each plate. All samples were run in duplicate. Results were analysed by 7500 software v.2.0.1 and GraphPad Prism (version 5.00 for Windows, Graph Pad Software, San Diego California USA, http://www.graphpad.com). The significance of differences between pre- and post-treatment groups was determined using paired Student's T test. Power calculations showed that N = 19 paired samples had 92.2% power to detect a difference in mean values pre- and post-treatment of 0.9 with standard deviation of 0.9 at alpha level 0.01; 98.2% power at alpha level 0.05.

## Results and Discussion

To test the hypothesis that polymorphisms at *CXCR1/CXCR2 *might influence susceptibility to VL in India we initially genotyped 3 tagging SNPs (Table 2) in 176 nuclear families (Table 1) used in our previous studies [19,21] that contain 313 offspring with VL collected in the area of Muzaffarpur, Bihar State, India, where *L. donovani *is endemic. Using the family-based association test (FBAT) [29,30] in this primary family dataset (Table 3) we found evidence (nominal *P*-values ≤ 0.03) for associations between VL and SNPs *CXCR1*_rs2234671 (Z-score = 2.935, *P *= 0.003) and *CXCR1*_rs3138060 (Z-score = 2.22, *P *= 0.026), but not with *CXCR2*_rs4674259. Since these two positive markers are in quite strong LD with each other (Additional File 2; Figure S2: D' = 0.84; r^2 ^= 0.46), these associations are likely to be measuring a single effect. The association at *CXCR1*_rs2234671 is robust to application of a strict Bonferroni correction for 3 independent (i.e. r^2 ^< 0.8) SNPs genotyped, which requires a significance cut-off of *P *≤ 0.017 (i.e. *P *= 0.05/3).

**Table 3:** Family-based association analysis between *CXCR1/CXCR2 *and VL

Common Designation	Allele	Allele frequency	# Fam	S	E(S)	Var(S)	Z	*P*
CXCR2_rs4674259	T	0.53	105	196	194	57.313	0.216	0.829

CXCR2_rs4674259	C	0.47	105	184	186	57.313	-0.216	0.829

**CXCR1_rs2234671**	G	0.85	69	181	165	31.519	2.935	**0.003**

**CXCR1_rs2234671**	C	0.15	69	61	77	31.519	-2.935	**0.003**

**CXCR1_rs3138060**	G	0.12	49	49	59	22.187	-2.222	**0.026**

**CXCR1_rs3138060**	C	0.88	49	133	122	22.187	2.222	**0.026**

Single point FBAT analysis under an additive model of inheritance for associations between *CXCR1/CXCR2 *polymorphisms and VL in the primary family-based sample set. # Fam = number of families informative for the FBAT analysis; S and E(S) represent the observed and expected transmissions for that allele, Var(S) is the variance. A positive Z score indicates association with disease; a negative Z score indicates the non-associated or protective allele or genotype. Bold indicates significant associations at nominal *P *≤ 0.05. The corrected *P*-value required to achieve significance taking account of 3 independent SNPs is *P *≤ 0.017.

**Figure 1:**
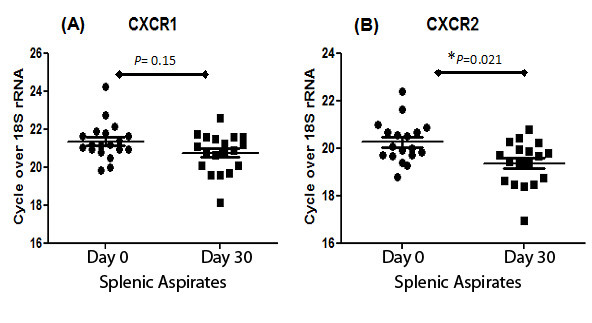
**Relative expression of CXCR1 (A) and CXCR2 (B) mRNA in paired splenic aspirates from VL patients before (Day 0) and after (Day 30) antileishmanial treatment**. Individual as well as mean (± SEM) relative expression for each group is indicated. Paired Student's t tests show significant differences in expression of CXCR2 but not CXCR1 when Day 0 values were compared to Day 30 values.

Evidence for an association between Indian VL and SNPs at *CXCR1*, which was consistent with data for CL/ML from Brazil [16], prompted us to pursue two further avenues of investigation. In so doing, we did not discount the possibility that *CXCR2 *might play a role, since the D' measure (Additional File 2; Figure S2) indicates strong LD across the *CXCR1/2 *SNPs for this sample (D' = 0.98 and 0.91). First, we looked at expression levels of CXCR1/CXCR2 in mRNA from splenic aspirates from 19 patients as paired samples taken pre- and post-treatment for VL (Figure 1). This demonstrated that CXCR2, but not CXCR1, is significantly (*P *= 0.021) reduced in expression in pre-treatment samples compared to post-treatment recovery samples, indicating that a deficiency in expression of CXCR2 might contribute to VL disease. Down regulation of CXCR2, but not CXCR1, was similarly observed in PMN from patients with pulmonary tuberculosis [31]. Secondly, we carried out a comprehensive replication of the association study in a much larger population-based case-control sample (Table 1) from the same region of Bihar State in India. Logistic regression analysis (Table 4) under an additive model of inheritance showed association between VL and SNPs *CXCR2*_rs4674259 (OR = 1.15, 95%CI = 1.01-1.31, *P *= 0.027) and *CXCR1*_rs3138060 (OR = 1.25, 95%CI = 1.02-1.53, *P *= 0.028), but not with *CXCR1*_rs2234671. Effect sizes (OR) are small, as is common in complex communicable or non-communicable diseases [32]. Significance was retained when either religion (*CXCR2*_rs4674259: OR = 1.15, 95%CI = 1.02-1.31, *P *= 0.028; *CXCR1*_rs3138060: OR = 1.26, 95%CI = 1.03-1.53, *P *= 0.026) or caste (*CXCR2*_rs4674259: OR = 1.15, 95%CI = 1.01-1.32, *P *= 0.039; *CXCR1*_rs3138060: OR = 1.25, 95%CI = 1.01-1.54, *P *= 0.037) was used as a covariate to take account of population substructure. Interestingly, although *CXCR1*_rs3138060 was the only SNP directly replicated across primary and replication samples, the same 3-SNP haplotype T_G_C (called on the negative strand; frequency 0.42) was shown to be the risk haplotype in both family- (Table 5; TRANSMIT; *P *= 0.014) and population- (Table 5; PLINK; OR = 1.16, *P *= 0.028) samples (combined *P *= 0.002). The 2- and 3-SNP over-transmitted haplotypes were more apparent in the family-based sample, which likely reflects over-relatedness for this set of families from this region of India where we showed previously that consanguineal marriages were common [19]. These haplotype results indicate that associations seen at *CXCR1 *might be due to LD with regulatory polymorphisms that influence *CXCR2 *expression. Together, the genetic and functional analyses favour a role for CXCR2 in contributing to susceptibility to VL in this region of India, although a role for CXCR1 cannot be discounted.

**Table 4:** Population-based association analysis between *CXCR1/CXCR2 *and VL

Common Designation	Allele	Affected	Unaffected	OR	L95	U95	*P-*values
**CXCR2_rs4674259**	T	**840/1036**	**816/1160**	**1.15**	**1.01**	**1.31**	**0.027**

CXCR1_rs2234671	G	286/1592	321/1657	0.92	0.77	1.10	0.384

**CXCR1_rs3138060**	C	**197/1685**	**252/1732**	**1.25**	**1.02**	**1.53**	**0.028**

Logistic regression analyses under an additive model for the replication sample of VL cases and controls. OR = odds ratio; L95 and U95 are lower and upper 95% confidence intervals. Allele counts are shown for minor/major allele for affected and unaffected individuals. Bold indicates associations significant at nominal *P *≤ 0.05. The corrected *P*-value required to achieve significance taking account of 3 independent SNPs is *P *≤ 0.017.

**Table 5:** Haplotype association analyses between *CXCR1/CXCR2 *and VL

Haplo	Family Analysis	Freq	CXCR2_rs4674259	CXCR1_rs2234671	CXCR1_rs3138060
T.G	TRANSMIT	0.41	χ^2 ^= 4.61; 1df; *P *= 0.032		

_ G.C	TRANSMIT	0.83		χ^2 ^= 10.27; 1df; *P *= 0.001	

T.G.C	TRANSMIT	0.40	χ^2 ^= 5.98; 1df; *P *= 0.014		

Haplo	Case-Control	Freq	CXCR2_rs4674259	CXCR1_rs2234671	CXCR1_rs3138060

T.G	PLINK	0.43	χ^2 ^= 4.34; 1df; *P *= 0.037		

_ G.C	PLINK	0.83		χ^2 ^= 1.29; 1df; *P *= 0.256	

T.G.C	PLINK	0.42	χ^2 ^= 4.99; 1df; *P *= 0.025		

Chi-squared (χ^2^), degrees of freedom (df) and *P*-values for risk associated (over-transmitted) haplotypes for the 3 SNPs as determined in TRANSMIT for the family-based primary sample, and in PLINK for the case-control replication sample. Bold indicates significant associations at nominal *P *≤ 0.05. Haplotypes are called on the negative strand across the 3 SNPs.

The involvement of either CXCR1 or CXCR2 in VL is of interest given recent observations on the different roles they may play in chemotaxis and activation of PMN in sites of infection [33], and the increasing recognition of the importance of PMN in the VL disease process [12,17]. Both are high affinity receptors for the CXC chemokine IL-8, but CXCR2 is more promiscuous in also binding a range of other CXC chemokines (CXCL-1, 2, 3, 5, 6, and 7) which may play important roles in directing a broader array of immune cells to sites of infection in the visceral organs. Recent studies [34] also show that, while neutralizing antibodies against CXCR2 abolish neutrophil extracellular trap (NET) formation, antibodies against CXCR1 have no effect. NETs are formed by DNA fibers decorated with antimicrobial proteins released from PMN upon activation. The release of DNA NETs, decorated with elastases and histones, by human PMN upon interaction with *Leishmania *parasites has been shown to ensnare the parasite and is leishmanicidal [10], adding to the potential importance of PMN in innate immunity to leishmanial infection.

Here we examined *CXCR1 *and *CXCR2 *as candidate genes for susceptibility to VL in India. Whilst SNPs at both loci were associated with VL, functional analysis of expression in splenic aspirates together with a common risk haplotype favour *CXCR2 *as the etiological gene regulating susceptibility to disease. Our data contribute to increasing evidence for an important role for PMN in directing the outcome of leishmanial infections in humans.

## Conclusions

From the results of this well-powered primary and replication genetic study, together with functional analysis of gene expression, we conclude that CXCR2 plays a role in determining outcome of VL in India.

## Competing interests

The authors declare that they have no competing interests.

## Authors' contributions

AM, MF and SM carried out the field collection and/or preparation of the samples. SM and JO performed the genotyping, and participated in the statistical analysis and interpretation of the data. MF cross-checked statistical analyses and carried out additional statistical tests, including the haplotype analyses. SEJ trained SM in the laboratory for genotyping techniques, in database entry and use of the genetic database GenIE in Perth, and in genetic statistical analysis methods. MR oversaw laboratory-based work in Varanasi. DSR and KT oversaw the Sequenom genotyping undertaken by SM in Hyderabad. MS and PT assisted with RNA preparation. SM designed and carried out the QRT/PCR. SS helped conceive the study, was responsible for clinical care of cases at the Kala Azar Medical Research Centre, Muzaffarpur, Bihar State, India, and provided the logistical support to make the study possible. SM prepared the first draft of the manuscript. JMB designed the study, conceived the specific hypothesis to be tested, made the final interpretation of the data, and prepared the final manuscript. All authors read and approved the final manuscript.

## Pre-publication history

The pre-publication history for this paper can be accessed here:

http://www.biomedcentral.com/1471-2350/12/162/prepub

## Supplementary Material

Additional file 1 **Figure S1**. Graphical representation of pairwise D' and r^2 ^LD measures across CXCR1 and CXCR2 in the HapMap CHB/JPT populations demonstrating large LD blocks tagged by SNPs *CXCR1*_rs3138060 and *CXCR2*_rs4674259 genotyped as tag-SNPs in the study. Click here for file

Additional file 2 **Figure S2**. Graphical representation of pairwise D' and r^2 ^LD measures across 3 SNPs genotyped in the study. Click here for file
