# Supplementary material for: Genetic and functional evaluation of the role of CXCR1 and CXCR2 in susceptibility to visceral leishmaniasis in north-east India
Source: BMC Med Genet. 2011 Dec 15;12:162. doi: 10.1186/1471-2350-12-162 (PMC3260103; doi:10.1186/1471-2350-12-162)
Supplement: Additional file 1 — Figure S1. Graphical representation of pairwise D' and r2 LD measures across CXCR1 and CXCR2 in the HapMap CHB/JPT populations demonstrating large LD blocks tagged by SNPs CXCR1_rs3138060 and CXCR2_rs4674259 genotyped as tag-SNPs in the study. [file 1471-2350-12-162-S1.PDF]

Additional File 1

Figure S1 - Graphical representation of pairwise D' and r^2 LD measures across CXCR1 and CXCR2 in the HapMap CHB/JPT populations demonstrating large LD blocks tagged by SNPs *CXCR1*_rs3138060 **and** *CXCR2*_rs4674259 **genotyped as tag-SNPs in the study.**
LD patterns for D' (above) and r^2 (below) were determined in Haploview software v4.2 [1] for all SNPs in the region with minor allele frequency >0.2. LD blocks were generated using the default Gabriel *et al.* algorithm [2]. SNP positions are shown relative to gene structure. A single SNP (blocked) could be chosen in each gene that tagged the whole gene from 5'UTR to 3'UTR or downstream region. D' values and confidence levels (LOD) are represented shades of pink for high D', LOD<2; white for D'<1, LOD<2. r^2 values are represented white for $r^2 = 0$, with intermediate values for $0<r^2< 1$ indicated by shades of grey. The numbers within the squares represent the D' or r^2 scores for pairwise LD.

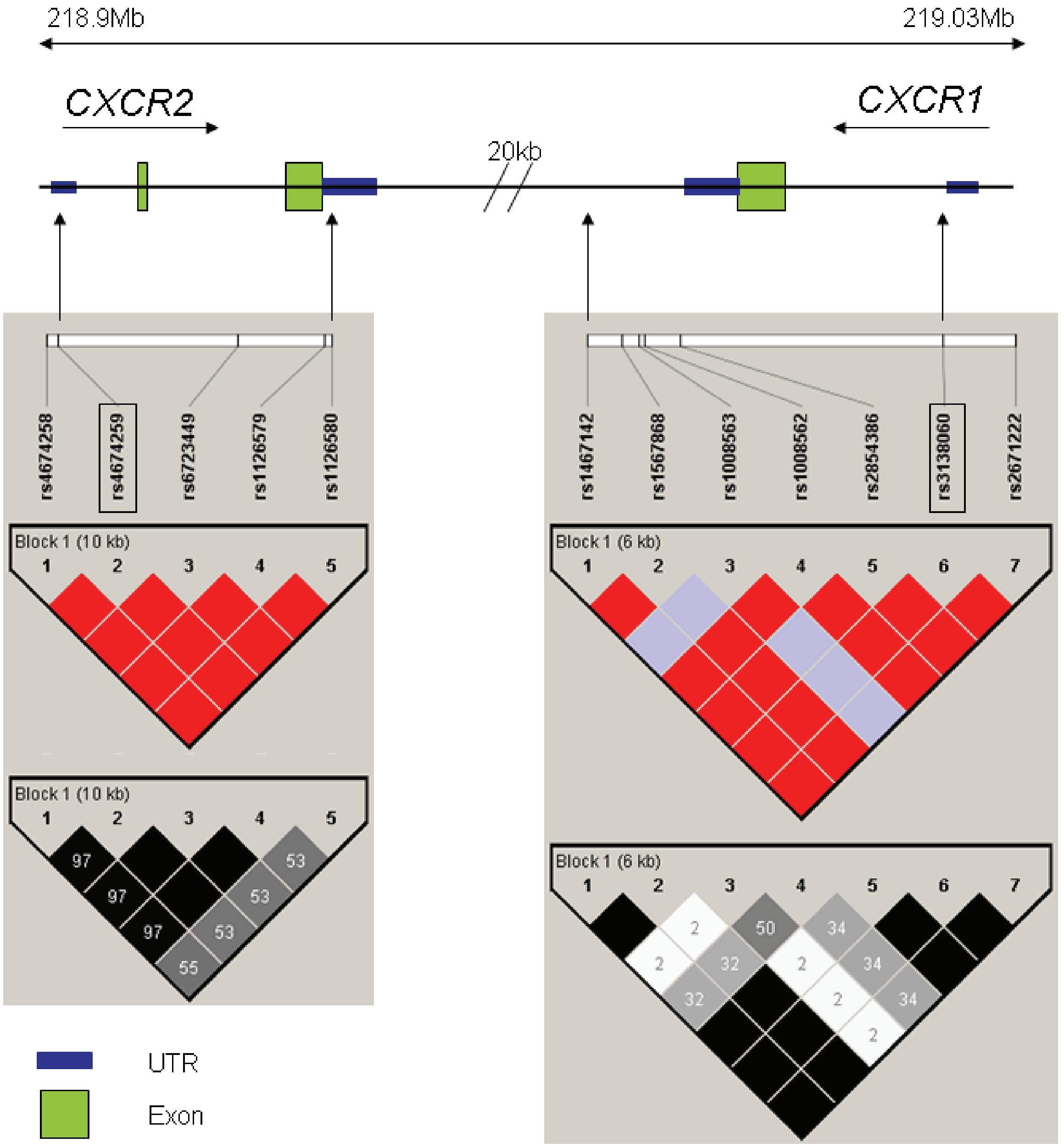

References

1. Barrett JC, Fry B, Maller J, Daly MJ: **Haploview: analysis and visualization of LD and haplotype maps**. *Bioinformatics* 2005, **21**(2):263-265.
2. Gabriel SB, Schaffner SF, Nguyen H, Moore JM, Roy J, Blumenstiel B, Higgins J, DeFelice M, Lochner A, Faggart M *et al*: **The structure of haplotype blocks in the human genome**. *Science* 2002, **296**(5576):2225-2229.
