# Supplementary material for: Genetic and functional evaluation of the role of CXCR1 and CXCR2 in susceptibility to visceral leishmaniasis in north-east India
Source: BMC Med Genet. 2011 Dec 15;12:162. doi: 10.1186/1471-2350-12-162 (PMC3260103; doi:10.1186/1471-2350-12-162)
Supplement: Additional file 2 — Figure S2. Graphical representation of pairwise D' and r2 LD measures across 3 SNPs genotyped in the study. [file 1471-2350-12-162-S2.PDF]

Additional File 2

Figure S2 - Graphical representation of pairwise D' and r^2 LD measures across 3 SNPs genotyped in the study.

LD patterns for D’ and r^2 were determined in Haploview software v4.2 [1]. The top panel shows LD values obtained using unrelated founders of the families used in the primary sample. The bottom panel shows LD values for controls used in the case-control analysis. D' values and confidence levels (LOD) are represented shades of pink for high D', LOD<2; white for D'<1, LOD<2. r^2 values are represented white for $r^2 = 0$, with intermediate values for $0<r^2< 1$ indicated by shades of grey. The numbers within the squares represent the D' or r^2 scores for pairwise LD.

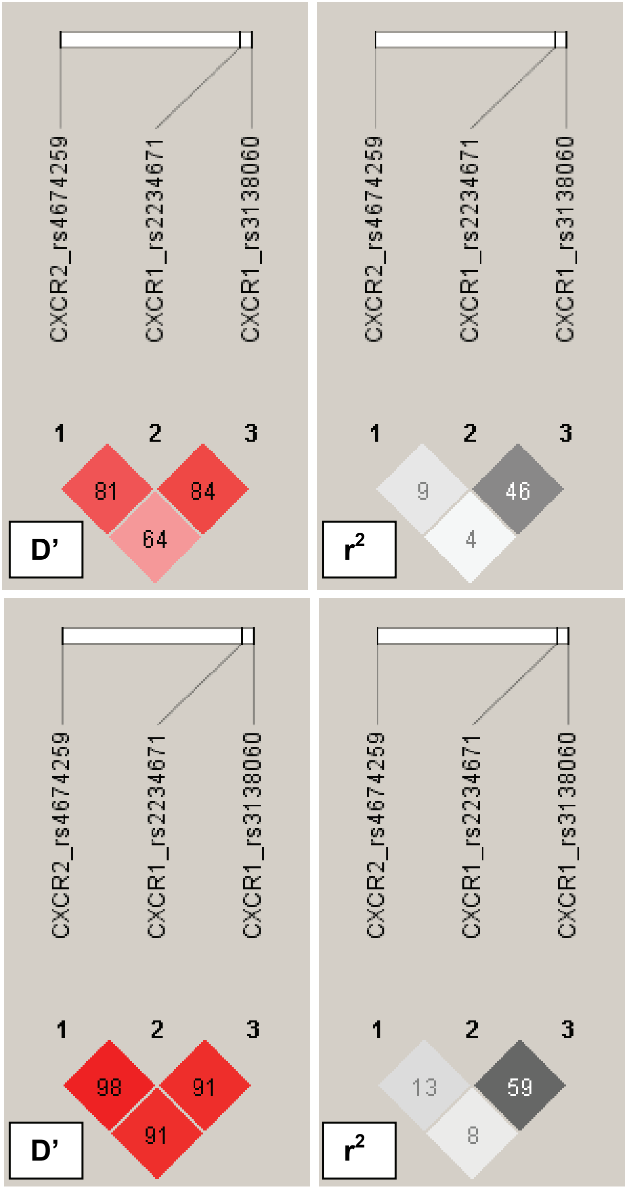

Reference

1. Barrett JC, Fry B, Maller J, Daly MJ: **Haploview: analysis and visualization of LD and haplotype maps**. *Bioinformatics* 2005, **21**(2):263-265.
